# Microbial carbon use efficiency and soil organic carbon: Which is the determinant?

**DOI:** 10.1016/j.xinn.2025.100984

**Published:** 2025-06-06

**Authors:** Bingchang Tan, Shiming Luo

**Affiliations:** 1 Agro-Environmental Protection Institute, Ministry of Agriculture and Rural Affairs, Tianjin 300191, China; 2 Institute of Tropical and Subtropical Ecology, South China Agricultural University, Guangzhou 510642, China

## Main text

Microorganisms play a critical role in mediating the soil organic carbon (SOC) turnover processes. Briefly, there are two pathways involved in the role of microorganisms in the processes.^1^^,^^2^ First, biosynthesis in microbial metabolism causes the accumulation of microbial necromass that favors SOC formation (e.g., the entombing effect [EE] pathway). Second, extracellular enzyme released from microorganisms boosts SOC decomposition (e.g., the decomposing effect [DE] pathway). These two critical but contrasting pathways spark important questions: which pathway dominates the role of microorganisms in SOC turnover and do microorganisms promote or inhibit SOC storage? Clarifying these issues is critical to both predicting SOC feedback to changing climate and enhancing SOC sequestration.

Microbial carbon use efficiency (CUE), defined as the ratio of the amount of C employed in new biomass relative to the amount of C that has been consumed, is an integrative metric that can capture the SOC turnover processes.^2^ According to the definition, high CUE is expected to increase microbial biomass, which potentially enhances both SOC formation and decomposition. With the evidence of an observed positive correlation between SOC and microbial CUE from 132 measurements, Tao et al.^2^ recently proposed that the EE pathway dominates the role of microorganisms in SOC turnover. Additionally, they reported a positive CUE-SOC relationship that emerged from a microbial model with 57,267 globally distributed vertical SOC profiles and suggested that microbial CUE is a determinant promoting global SOC storage.

Here, we provide new insight into the role of microorganisms in SOC turnover processes and suggest that the DE dominates the role of microorganisms in SOC turnover and that microbial CUE inhibits SOC storage.

## Observed effect of SOC on microbial CUE

**Figure 1:**
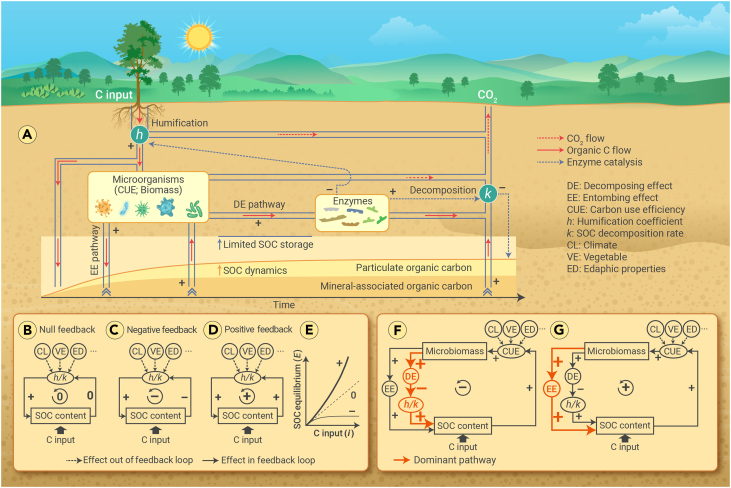
SOC turnover processes and the SOC turnover feedback loops (A) Sufficient exogenous C input increases SOC storage. Through humification (*h*), exogenous C (e.g., from plant) is divided into three flows: release to the atmosphere as CO_2_, be incorporated into SOC directly, and be absorbed by microorganisms. Microorganisms absorb C from exogenous C and SOC, generate necromass to form SOC (e.g., entombing effect [EE] pathway), and release enzymes to boost the decomposition of SOC (higher *k*) and exogenous C (lower *h*), thereby triggering SOC loss (e.g., decomposing effect [DE] pathway). Microorganisms also release CO_2_ via respiration. As SOC content accumulates over time, its decomposability increases simultaneously, because the proportion of active SOC (e.g., particulate organic carbon) rises. This makes SOC-derived energy and nutrients more accessible for microorganisms and increases microbial CUE and biomass, which both enhance SOC formation and decomposition. (B–E) Owing to the different effects (null, negative, and positive) of SOC on the *h/k* ratio, there is null (B), negative (C), and positive (D) feedback of SOC turnover, which leads to a linear, asymptotic, and exponential *E*-*i* relationship, respectively (E). The negative feedback (C) is most likely to occur since the soil’s capacity to store C is finite. (F) The DE pathway that decreases the *h/k* ratio outweighs the EE pathway, causing negative feedback. (G) If the EE pathway were to outweigh the DE pathway, positive feedback would be formed. Factors such as climate (CL), vegetation (VE), and edaphic properties (EDs) can affect the SOC turnover processes, but these factors and C input are not included in a feedback loop since they are independent of SOC content. Symbols “–”, “+”, and “0” indicates inhibition, promotion, and null effect, respectively.

SOC is observed to be positively correlated with microbial CUE.^2^ Consequently, microbial CUE is suggested to promote SOC storage, mainly because high CUE leads to high microbiomass and the accompanying production of microbial necromass.^1^^,^^2^ While CUE is considered an independent variable affecting SOC content,^2^ the potential effect of SOC on CUE is neglected. Here, we suggest that SOC promotes microbial CUE. Firstly, the measured SOC should be considered as an independent variable affecting CUE. Litter or root in soil samples, which are used to simultaneously measure SOC and CUE, are removed before the measurements; hence, SOC is the only source of energy and nutrients for microbial metabolism. In this case, microorganisms cannot boost SOC storage without exogenous C input, but they are still able to survive by relying on the support of SOC and rapidly respond to the variations in SOC content. Therefore, the measured CUE-SOC relationship does not necessarily reveal how microbial CUE affects SOC. Instead, SOC should be considered an independent variable affecting CUE. Secondly, the enhancement of microbial CUE by SOC is logical, although there are still large uncertainties on the CUE-SOC relationship (null, positive, or negative) due to different estimation methods (e.g., ^13/14^C labeling, ^18^O H_2_O, and stoichiometric models) and various factors (e.g., across various temporal, spatial, and biological scales).^3^ The capacity of soil to protect SOC (e.g., via mineral adsorption) is finite. As SOC accumulates, its decomposability increases because the proportion of unprotected SOC (e.g., particulate organic carbon) rises (Figure 1A),^4^ making SOC-derived energy and nutrients more accessible to microorganisms. This enables microorganisms to allocate fewer resources to energy and nutrient acquisition, thereby enhancing CUE and biomass production (Figure 1A). Therefore, the observed positive CUE-SOC relationship should be interpreted as evidence supporting the promotion of microbial CUE by SOC rather than the reverse.

## Negative feedback in SOC turnover processes

Given the observed promotion of CUE by SOC, we propose that the interaction (i.e., feedback) between SOC and CUE should be considered when studying the role of microorganisms on SOC turnover.

At the system level, SOC dynamics is the result of the balance between SOC formation and decomposition (Equation 1).

(Equation 1) $$\frac{dX}{dt}=ih-Xk\text{,}$$

where *X* is SOC content (g kg^−1^), *t* is time (year), *i* is exogenous C input level per soil mass (e.g., from plant litter and root, g kg^−1^ year^−1^), *h* is humification coefficient (unitless) of input organic materials that reflects the proportion of input C incorporated into SOC, and *k* is SOC decomposition rate (year^−1^). High *h* benefits SOC formation, whereas high *k* boosts SOC loss.

According to Equation 1, when SOC is at equilibrium (*E*, g kg^−1^), *dX/dt* = 0; thus,

(Equation 2) $$E=X=\frac{h}{k}i\text{.}$$

Equation 2 indicates that the *h/k* ratio is essential for determining SOC storage: a higher *h/k* ratio indicates greater SOC storage. Climate, vegetation, and edaphic properties are important factors influencing SOC turnover.^2^ Hence, these three factors can influence the *h/k* ratio. When these factors are relatively stable (e.g., at a specific site), the *h/k* ratio is expected to be constant if it is not affected by SOC content (Figure 1B). As a result, SOC equilibrium would linearly increase with C inputs (linear *E*-*i* relationship, line “0” in Figure 1E) according to Equation 2. However, SOC cannot increase without limit (i.e., SOC saturation),^4^^,^^5^ and SOC equilibrium is expected to asymptotically increase to a maximum value with C inputs (asymptotic *E*-*i* relationship).^4^ This asymptotic *E*-*i* relationship can be attributed to negative feedback mechanisms in SOC turnover processes.^4^ Specifically, an increase in SOC content reduces the *h/k* ratio (Figure 1C), which in turn causes SOC equilibrium to asymptotically increase with C inputs (curve “–” in Figure 1E) according to Equation 2. Conversely, if SOC were to enhance the *h/k* ratio (Figure 1D), SOC equilibrium would exponentially increase with C inputs (exponential *E*-*i* relationship) (curve “+” in Figure 1E). Given the soil’s finite ability to store C, negative feedback—rather than null or positive feedback—is expected to occur in SOC turnover processes.

Like *h* and *k*, microorganisms play a crucial role in mediating SOC turnover processes, not only for their role in SOC formation and decomposition but also for their response to changes in the soil environment, including varying SOC content. Hence, the negative feedback between CUE and SOC also works via modifying *h* and *k*.

## Microbial CUE inhibits SOC storage

As discussed above, the soil’s finite ability to store C implies a negative feedback loop between SOC and CUE; meanwhile, SOC promotes microbial CUE. Therefore, there should be a negative effect of microbial CUE on SOC storage; specifically, the DE pathway, which enhances SOC loss, dominates the role of microorganisms in SOC turnover (Figure 1F). The enhanced extracellular enzyme production boosts the decomposition of SOC (i.e., higher *k*) and exogenous C, leaving a lower proportion of exogenous C to form SOC (i.e., lower *h*) (Figure 1A). These processes reduce the *h/k* ratio with rising SOC content (Figure 1F), finally causing an asymptotic *E*-*i* relationship (curve “–” in Figure 1E).

Tao et al.^2^ additionally reported a positive CUE-SOC relationship using their microbial model with 57,267 globally distributed vertical SOC profiles. We posit that this outcome depends on the model structure and parameters. Although data assimilation in their study facilitates parameter optimization, their microbial model does not consider the effect of SOC on CUE. Given the positive effect of SOC on CUE, if CUE were to promote SOC storage—meaning that the EE pathway outweighs the DE pathway—it would create positive feedback of SOC turnover (Figure 1G), consequently leading SOC equilibrium to infinitely increase with C input in an exponential pattern (curve “+” in Figure 1E), which is unlikely to happen. Hence, we propose that microbial CUE inhibits SOC storage rather than promoting it.

Beyond theoretically demonstrating the hypotheses of a dominant microbial effect (Figures 1F and 1G), the SOC sequestration rate (*dX/dt* in Equation 1) can be employed to compare the relative contributions of DE and EE pathways. Notably, the *dX/dt* > 0 in Equation 1 should not be misinterpreted as the EE pathway outweighing the DE pathway since whether SOC increases or decreases is determined by the exogenous C input level and the initial SOC content rather than soil microorganisms. Under continuous sufficient C input, SOC asymptotically approaches equilibrium over time, implying that the SOC sequestration rate declines to zero. Conversely, microbial CUE and biomass rise (Figure 1A). These inverse trends imply that the rising overall effect of microorganisms inhibits soil C sequestration. The higher the SOC content, the more significantly the overall effect inhibits SOC sequestration. In other words, the DE pathway should dominate the role of microorganisms in SOC turnover processes. Conversely, if the EE pathway were to outweigh the DE pathway, then SOC-rich soils should sequester SOC more rapidly than SOC-poor soils. However, it is widely reported that SOC-rich soils exhibit weaker gains or stronger losses of SOC than SOC-poor soils. Therefore, high CUE, despite the subsequent high microbial necromass, should inhibit SOC storage.

## Conclusion

Microorganisms play a critical role in mediating SOC turnover. We suggest that an observed positive CUE-SOC relationship does not indicate a promotion of SOC storage by CUE. Instead, SOC promotes microbial CUE, and the enhanced CUE in turn inhibits SOC storage. In other words, the DE is expected to outweigh the EE and dominate the role of microorganisms in SOC turnover. The negative feedback between microorganisms and SOC should be considered in future research on SOC turnover, including developments in CUE observation techniques and SOC modeling.

## Funding and acknowledgments

This work was supported by the 10.13039/501100012166National Key Research and Development Program of China (2023YFD1701803) and the 10.13039/501100012421Agricultural Science and Technology Innovation Program of China (AEPI).

## Declaration of interests

The authors declare no competing interests.
